# Synchrony of nitrogen supply and crop demand are driven via high maize density in maize/pea strip intercropping

**DOI:** 10.1038/s41598-019-47554-1

**Published:** 2019-07-29

**Authors:** Zhilong Fan, Yanhua Zhao, Qiang Chai, Cai Zhao, Aizhong Yu, Jeffrey A. Coulter, Yantai Gan, Weidong Cao

**Affiliations:** 1 Gansu Provincial Key Laboratory of Arid land Crop Science, Lanzhou, 730070 China; 2 The Faculty of Agronomy, Gansu Agricultural University, Lanzhou, 730070 China; 3 College of Resources and Environmental Sciences, Gansu Agricultural University, Lanzhou, 730070 China; 4 Department of Agronomy and Plant Genetics, University of Minnesota, St. Paul, MN 55108 USA; 5 Agriculture and Agri-Food Canada, Swift Current, SK S9H 3X2 Canada; 6 Key Laboratory of Plant Nutrition and Fertilizer, Ministry of Agriculture and Rural Affairs/Institute of Agricultural Resources and Regional Planning, Chinese Academy of Agricultural Sciences, Beijing, 100081 China

**Keywords:** Agroecology, Agroecology

## Abstract

Cereal density may influence the balance between nitrogen (N) supply and crop N demand in cereal/legume intercrop systems. The effect of maize (*Zea mays* L.) plant density on N utilization and N fertilizer supply in maize/pea (*Pisum sativum* L.) strip intercropping was evaluated in a field study with sole maize, sole pea, and intercropped maize/pea with three maize densities (D1, 45,000 plants ha^−1^; D2, 52,500 plants ha^−1^; D3, 60,000 plants ha^−1^) and two N treatments (N0, 0 kg N ha^−1^; N1, 450 kg N ha^−1^ for maize and 225 kg N ha^−1^ for pea). Soil mineral N in intercropped strips decreased with increased maize density. Increased maize density decreased N accumulation for intercropped pea but increased it for maize and the sum of both intercrops. The land equivalent ratio for grain yield (LER grain) showed a 24–30% advantage for intercrops than corresponding sole crops, and was greater with D3 than D1 and D2. Maize/pea intercropping had 4–113% greater nitrogen use efficiency (NUE) than sole maize, which was enhanced with increased maize density. Increasing maize density improved the synchrony of N supply and crop demand in maize/pea strip intercropping.

## Introduction

Nitrogen (N) is a major plant nutrient, that is typically the most limiting in agriculture and plays a crucial role in the proper functioning of cropping systems^1,2^. Synthetic fertilizers and biological N_2_ fixation by legumes are the most important sources of N^3,4^. Biological N_2_ fixation by legumes is more renewable and environmentally friendly than application of N fertilizer^5^. In general, legume-based cropping systems are more sustainable than fertilizer-based systems^6^. Cereal/legume intercropping systems are utilized worldwide for food and feed production due to the facilitative interactions of intercrops and biological N_2_ fixation^7^. The substantial complementarities in the use of N resources between intercropped legumes and cereals has been verified in several studies^8–10^. Cereal/legume intercropping may sustain the yield of both crops under low N application^11^. Unfortunately, in China, intercropping has been developed using intensive farming systems with high levels of inputs, such as N fertilizer, to produce high yields^12^. The high levels of inputs with inefficient N use result in a large portion of the N applied being lost to the environment through gaseous losses and leaching^13^.

A major cause of inefficient use of N fertilizer and many environmental hazards associated with excess N in the biosphere is the asynchrony between the N supply and crop demand^6^. Fertilizer N use is inefficient in most cropping systems, with only about half of the applied N taken up by crops during the growing season^14^. The uncertainty faced by famers in determining fertilizer application rates includes not knowing the existing supply of N available in the soil as well as not being able to predict the N demand of the crop^15^. Soil N supply is often ignored when fertilizer rates are determined^16^. Fertilizer rates should be based on eliminating uncertainties in soil N supply and crop N demand. Eliminating the uncertainty of the soil N supply would reduce average N rates by 20–40% in maize (*Zea mays* L.), while perfect knowledge of potential crop N demands would reduce N rates by 3–10%, and combined knowledge of both factors would reduce N rates significantly more than the sum of their individual effects^16^. Studies on improving N use efficiency (NUE) of cereal/legume intercropping systems have primarily focused on certain specific interspecific interactions, N fertilization rates, and N management^17^, irrespective of the relationship between soil N supply and crop N demand. Strategies should be taken to improve the synchrony of N supply and demand in farming systems, thereby increasing NUE.

Plant density can be used as a regulator for specific purposes, such as achieving high N yield^18^. Legumes under severe competition from maize show more efficient use of scarce resources, such as available soil N^19–21^. Additionally, greater plant density of the cereal may enhance its competition relative to the legume in intercropping systems^22^. The greater demand of cereal intercrops for soil inorganic N may force the legume to rely on N_2_ fixation, and enhance the balance between crop N demand and N supply. Understanding the response of soil mineral N and the balance between N supply and crop N demand as affected by plant density of the cereal is vital to maximizing grain yield and NUE of cereal/legume strip intercropping systems. We hypothesized that in a maize/pea (*Pisum sativum* L.) strip intercropping system, (i) the effect of maize plant density on soil mineral N could improve symbiotic N fixation of pea and increase N accumulation and yield of the intercrops, and (ii) the synchrony between crop N demand and N supply (via fertilizer applications or organic matter mineralization) with excessive N fertilizer inputs would be improved with maize at a greater plant density. Therefore, the following were measured: (i) soil mineral N variation in intercropping and sole cropping systems during the growing season, (ii) symbiotic N fixation of pea and N accumulation of component intercrops, (iii) the balance between N demand and supply in intercropping and sole cropping systems, and (iv) yield and N use efficiency of pea and maize in intercropping and sole cropping systems.

## Results

### Soil mineral N

**Figure 1:**
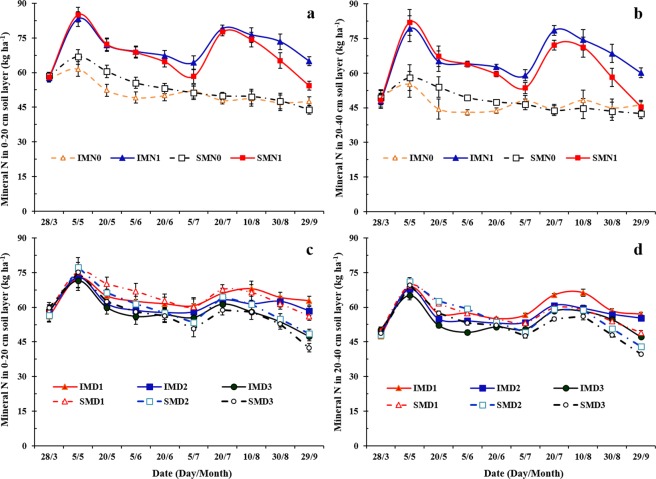
Mineral nitrogen (N) (ammonium-N + nitrate-N) in the 0–20 cm soil layer (**a**,**c**) and 20–40 cm soil layer (**b**,**d**) of intercropped maize (IM) or sole maize (SM) with two N treatments (N0, 0 kg N ha^−1^; and N1, 225 kg N ha^−1^ for pea and 450 kg N ha^−1^ for maize) (**a**,**c**) and three maize densities (D1, 73,600 plants ha^−1^; D2, 85,900 plants ha^−1^; and D3, 98,200 plants ha^−1^ for sole maize; the relative density of intercropped maize (according to the proportion of occupied area in intercropping) was 45,000, 52,500, and 60,000 plants ha^−1^ for D1, D2, and D3, respectively) (**b**,**d**). Error bars indicate standard errors of the means (*n* = 4 for (**a**,**b**); *n* = 6 for (**c**,**d**)).

Greater soil mineral N was observed in the intercropped maize strips than that of sole maize at certain growth stages (Fig. 1). In the 0–20 cm soil layer, mineral N of intercropped maize strips was greater than that of sole maize by an average of 10, 13, and 20% on July 5 (the pea harvest date), August 30, and September 29, respectively. In the 20–40 cm soil layer, mineral N of intercropped maize strips averaged 5–33% greater than that of sole maize from July 5 to September 29 (Fig. 1a,b). Compared to sole maize with N application, soil mineral N content in intercropped maize strips with N application was 33 and 36% greater in the 0–20 cm and 20–40 cm layers, respectively. Soil mineral N of sole and intercropped maize strips decreased with increasing maize plant density (Fig. 1c,d). Soil mineral N content in maize strips decreased by 6 and 5% in the 0–20 and 20–40 cm soil layers, respectively, as maize plant density increased from D1 (45,000 plants ha^−1^) to D2 (52,500 plants ha^−1^); soil mineral N in maize strips decreased by 3.3 and 4.9% in the 0–20 and 20–40 cm layers, respectively, as maize density increased from D2 to D3 (60,000 plants ha^−1^).

**Figure 2:**
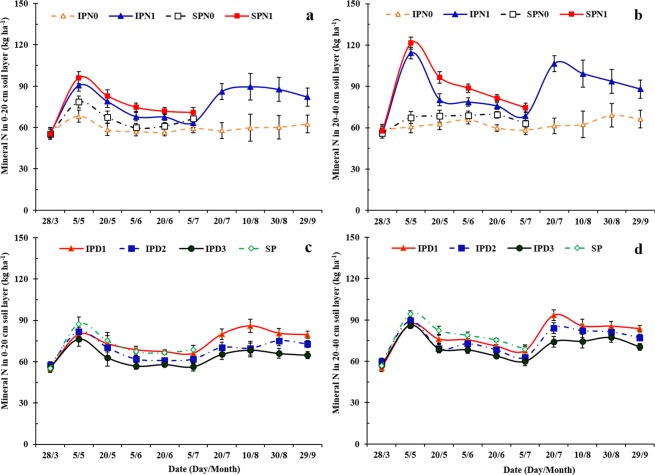
Mineral nitrogen (N) (ammonium-N + nitrate-N) in the 0–20 cm soil layer (**a**,**c**) and 20–40 cm soil layer (**b**,**d**) of intercropped pea (IP) or sole pea (SP) with two N treatments (N0, 0 kg N ha^−1^; and N1, 225 kg N ha^−1^ for pea and 450 kg N ha^−1^ for maize) (**a**,**c**) and three maize densities in intercropping (D1, 73,600 plants ha^−1^; D2, 85,900 plants ha^−1^; and D3, 98,200 plants ha^−1^ for sole maize; the relative density of intercropped maize (according to the proportion of occupied area in intercropping) was 45,000, 52,500, and 60,000 plants ha^−1^ for D1, D2, and D3, respectively) (**b**,**d**). Error bars indicate standard errors of the means (*n* = 4).

Soil mineral N content of pea strips changed substantially during the growing season (Fig. 2), with no significant year-by-treatment interactions observed. During the co-growth period (before pea harvest), intercropped pea strips had less soil mineral N compared to that of sole pea (Fig. 2a,b). From 5 May to 5 July, mineral N in the 0–20 and 20–40 cm soil layers of intercropped pea was 6.8–10.6% and 7.7–12.6% less than that of sole pea, respectively. Soil mineral N of intercropped pea increased with the application of N; this was most notable during the later part of the growing season of maize, as well as after pea harvest (from 20 July to 29 September). Mineral N content in intercropped pea strips with N application (IPN1) was 28.7 and 37.6% greater than that in the intercropped pea without N application (IPN0) for the 0–20 cm and 20–40 cm soil layers, respectively. Increasing maize plant density reduced the soil mineral N of intercropped pea strips during the growing period from 20 May to 29 September (Fig. 2c,d). Mineral N in the 0–20 and 20–40 cm soil layers of the intercropped pea strips decreased by 3.9–18.9% and 3.6–9.9%, respectively, when maize density increased from D1 to D2; it decreased by 4.9–12.3% and 4.5–11.8% in the 0–20 and 20–40 cm soil layers, respectively, when maize density increased from D2 to D3.

**Figure 3:**
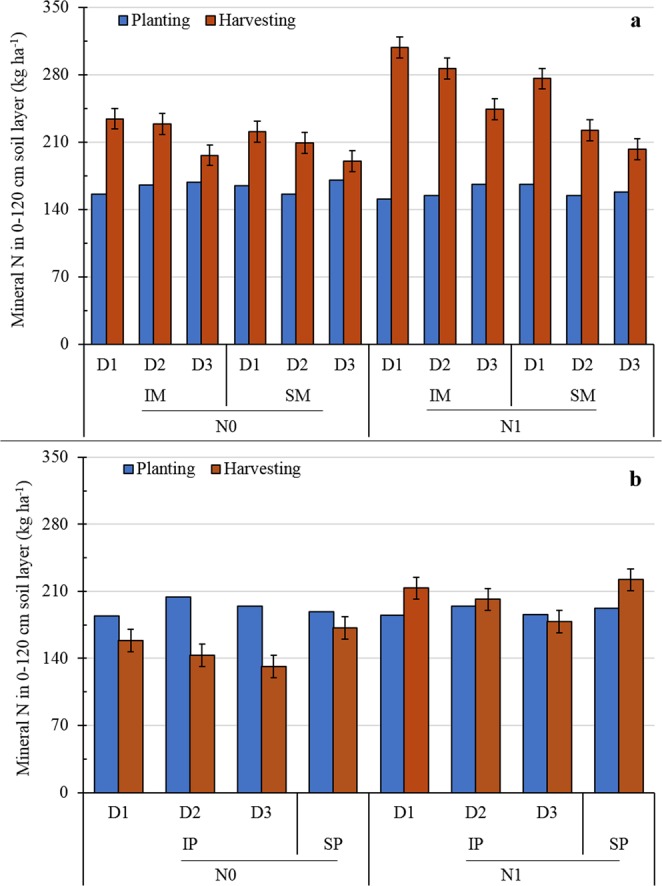
Mineral nitrogen (N) (ammonium-N + nitrate-N) in the 0–120 cm soil layer of intercropped pea (IP) or sole pea (SP) (**a**) and intercropped maize (IM) or sole maize (SM) (**b**) with two N treatments (N0, 0 kg N ha^−1^; and N1, 225 kg N ha^−1^ for pea and 450 kg N ha^−1^ for maize) (**b**) and three maize densities in intercropping (D1, 73,600 plants ha^−1^; D2, 85,900 plants ha^−1^; and D3, 98,200 plants ha^−1^ for sole maize; the relative density of intercropped maize (according to the proportion of occupied area in intercropping) was 45,000, 52,500, and 60,000 plants ha^−1^ for D1, D2, and D3, respectively) (**b**,**d**). Error bars indicate standard errors of the means (*n* = 4).

Compared to sole maize, intercropped maize strips had 14% greater mineral N content in the 0–120 cm soil layers at maize harvest (Fig. 3a). The application of N significantly increased soil mineral N content in both intercropped and sole maize strips. Mineral N content in the 0–120 cm soil layer of maize strips with N application (N1) was 20% greater than that without N application (N0). Similar to pea, mineral N content in the 0–120 cm soil layer of intercropped and sole maize strips decreased by 9 and 12% as maize plant density increased from D1 to D2 and from D2 to D3, respectively. At pea harvest, total mineral N content in the 0–120 cm soil layer of pea strips differed significantly among treatments, with no significant year-by-treatment interactions (Fig. 3b). Intercropped pea strips had 13% less mineral N in the 0–120 cm soil layer than sole pea. Compared to N0, N1 significantly increased mineral N in the 0–120 cm soil layer of pea. Increasing maize plant density decreased soil mineral N content of intercropped pea strips. Soil mineral N content of intercropped pea strips was reduced by 7 and 10% as maize plant density increased from D1 to D2 and from D2 to D3, respectively.

### Crop N accumulation

Cropping system, N rate, and maize plant density significantly influenced total N accumulation in maize (Table 1). Total N accumulation of intercropped maize reached 76% of that of sole maize. Nitrogen application significantly increased total N accumulation of maize compared to N0. On average, total N accumulation of maize increased by 16 and 8% when maize plant density increased from D1 to D2 and from D2 to D3, respectively. The response of maize seed N to cropping system, N rate, and maize planting density was similar to that of total N accumulation. Seed N accounted for 66% of the total N accumulation by maize, which was increased by 7% when intercropped with pea.

**Table 1:** Seed N, straw N and total N accumulation of maize and pea planted in intercropping or sole cropping, with two N treatments (N0, 0 kg N ha^−1^; and N1, 225 kg N ha^−1^ for pea and 450 kg N ha^−1^ for maize) and three maize densities (D1, 73,600 plants ha^−1^; D2, 85,900 plants ha^−1^; and D3, 98,200 plants ha^−1^ for sole maize; the relative density of intercropped maize (according to the proportion of occupied area in intercropping) was 45,000, 52,500, and 60,000 plants ha^−1^ for D1, D2, and D3, respectively)^b^.

Effect	Maize			Pea		
	Seed N	Straw N	Total N accumulation	Seed N	Straw N	Total N accumulation
	kg ha^−1^					
Year						
2012	150	43	193	87	64	151
2013	107	96	202	84	45	129
2014	135	75	210	79	62	142
LSD_(0.05)_	56	59	37	16	35	39
Cropping system						
Intercropping	118	57	175	70	46	116
Sole cropping	143	86	229	124	89	214
LSD_(0.05)_	43	21	99	37	41	73
Nitrogen rate						
N_0_	108	38	146	74	50	124
N_1_	153	105	257	93	64	157
LSD_(0.05)_	34	52	57	12	11	24
Maize density						
D_1_	114	64	178	70	57	127
D_2_	132	74	206	73	48	121
D_3_	146	77	222	67	34	101
LSD_(0.05)_	16	7	18	5	10	15
	*P* > *F*					
Year (Y)	NS^a^	NS	NS	NS	NS	NS
Cropping system (C)	0	0.019	0.002	0.001	0.004	0.007
Nitrogen rate (N)	0	0.001	0.013	0.011	0.001	0.001
Maize density (D)	0.002	0.041	0.029	0.021	0.013	0.033
C × N	NS	NS	NS	NS	NS	NS
N × D	NS	NS	NS	NS	NS	NS
D × C	NS	NS	0.039	NS	NS	NS

^a^NS, not significant at the 0.05 probability level. ^b^Values are means (*n* = 3).

Total N accumulation of intercropped pea reached 54% of that of sole pea (Table 1). Total N accumulation of intercropped pea with N application (N1) was 30% greater than that of pea with N0. Increasing maize plant density reduced total N accumulation of intercropped pea. The D3 intercropped pea exhibited 20% less total N accumulation than that of D1 and 16% less than that of D2. The effect of cropping system, N rate, and maize plant density on pea seed N was similar to that of total N accumulation of pea. Seed N accounted for 60% of the total N accumulation by pea. Increasing maize plant density increased the ratio of seed N to total N accumulation of intercropped pea. Seed N represented 54, 60, and 66% of total N accumulation of pea for D1, D2, and D3, respectively.

Total N accumulation of intercrops was significantly greater than that of the corresponding sole crops (Table 2). With N application, total aboveground N accumulation of maize/pea intercrops was 8–12% greater than that of sole maize and 34–53% greater than that of sole pea. The interaction between N treatment and maize plant density significantly influenced total N accumulation of intercrops. Without N application, total N accumulation of intercrops was not affected by maize plant density. With N application (N1, 225 kg N ha^−1^ for pea and 450 kg N ha^−1^ for maize), total N accumulation of intercrops increased by 10 and 4% as maize plant density increased from D1 to D2 and from D2 to D3, respectively.

**Table 2:** Nitrogen (N) accumulation of intercrops relative to corresponding sole crops with two N treatments (N0, 0 kg N ha^−1^; and N1, 225 kg N ha^−1^ for pea and 450 kg N ha^−1^ for maize) and three maize densities (D1, 73,600 plants ha^−1^; D2, 85,900 plants ha^−1^; and D3, 98,200 plants ha^−1^ for sole maize; the relative density of intercropped maize (according to the proportion of occupied area in intercropping) was 45,000, 52,500, and 60,000 plants ha^−1^ for D1, D2, and D3, respectively)^c^.

Nitrogen rates	Maize density	N accumulation (kg ha^−1^)			N equivalent ratio (NER)		
		Sole pea	Sole maize	Intercrops	Pea	Maize	Total
N0	D1	198	161	205	0.55 ± 0.02^a^	0.60 ± 0.05	1.16 ± 0.07
	D2		181	214	0.54 ± 0.04	0.61 ± 0.06	1.15 ± 0.04
	D3		214	204	0.46 ± 0.03	0.54 ± 0.02	1.00 ± 0.05
N1	D1	230	281	308	0.61 ± 0.02	0.61 ± 0.03	1.22 ± 0.03
	D2		313	339	0.59 ± 0.02	0.66 ± 0.02	1.26 ± 0.02
	D3		314	353	0.56 ± 0.05	0.73 ± 0.04	1.29 ± 0.03
LSD_(0.05)_		13.7					
				*P* > *F*			
Nitrogen rate (N)				0.000	0.001	0.000	0.034
Maize density (D)				0.013	0.019	0.021	0.023
L × D				0.029	NS^b^	0.035	0.042

^a^Values following ± are standard errors. ^b^NS, not significant at the 0.05 probability level. ^c^Values are means (*n* = 6).

There was a marked advantage of intercropping for crop N accumulation, as the total N equivalent ratio (NER) for maize/pea intercrops ranged from 1.00 to 1.29 (Table 2). The NER of maize/pea intercrops with N application was 5–26% greater than that with N0. For the N1 treatment, the advantage of intercropping on crop N accumulation was enhanced with increased maize plant density; the NER increased by 3 and 2% as maize planting density increased from D1 to D2 and from D2 to D3, respectively. The advantage of intercropping on maize N accumulation was influenced by the interaction between N treatment and maize plant density. With N0, the partial NER for maize in the D3 treatment was 10 and 11% less than that of maize in the D1 and D2 treatments, respectively. For N1, partial NER for maize increased by 8 and 10% as maize plant density increased from D1 to D2 and from D2 to D3, respectively. The advantage of intercropping for N accumulation of pea was diminished in D3 compared with D1 and D2. The partial NER for pea in the D3 treatment was 12 and 10% less than that of pea in the D1 and D2 treatments, respectively.

### N fixation and %N_dfa_ of pea

**Figure 4:**
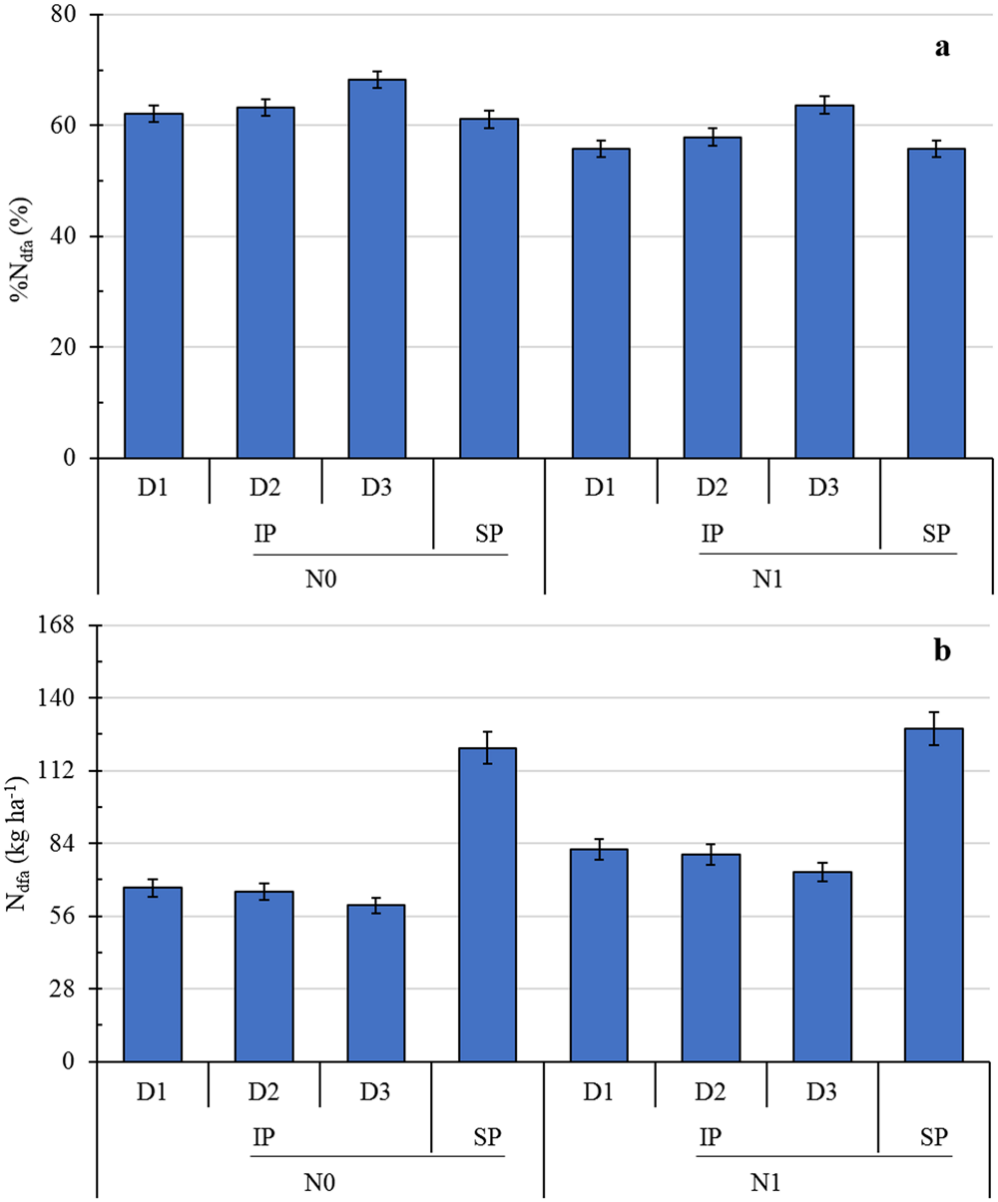
The percentage of total aboveground N accumulation derived from N_2_ fixation (%N_dfa_) (**a**) and the calculation N derived from the atmosphere (N_dfa_) (**b**) of intercropped pea (IP) or sole pea (SP), with two N treatments (N0, 0 kg N ha^−1^; and N1, 225 kg N ha^−1^ for pea and 450 kg N ha^−1^ for maize) and three maize densities (D1, 73,600 plants ha^−1^; D2, 85,900 plants ha^−1^; and D3, 98,200 plants ha^−1^ for sole maize; the relative density of intercropped maize (according to the proportion of occupied area in intercropping) was 45,000, 52,500, and 60,000 plants ha^−1^ for D1, D2, and D3, respectively).

The percentage of total aboveground N accumulation for pea, derived from N_2_-fixation (%N_dfa_) did not differ between intercropped pea and sole pea (Fig. 4a). The %N_dfa_ of intercropped and sole pea was 9% less with N1 compared to N0. The %N_dfa_ of intercropped pea increased with increasing maize planting density. The %N_dfa_ of intercropped pea in treatment D3 was 2 and 9% greater than that of pea in D1 and D2, respectively.

Nitrogen derived from the atmosphere (N_dfa_) of intercropped pea reached 57% of that of sole pea (Fig. 4b). The N_dfa_ of pea was significantly enhanced by N application. The N_dfa_ of pea with 225 kg ha^−1^ N application (N1) was 18% greater than that in treatment N0. The N_dfa_ of intercropped pea in treatment D3 was 11 and 8% less than that of pea in D1 and D2, respectively.

### Balance between crop N demand and N supply

**Figure 5:**
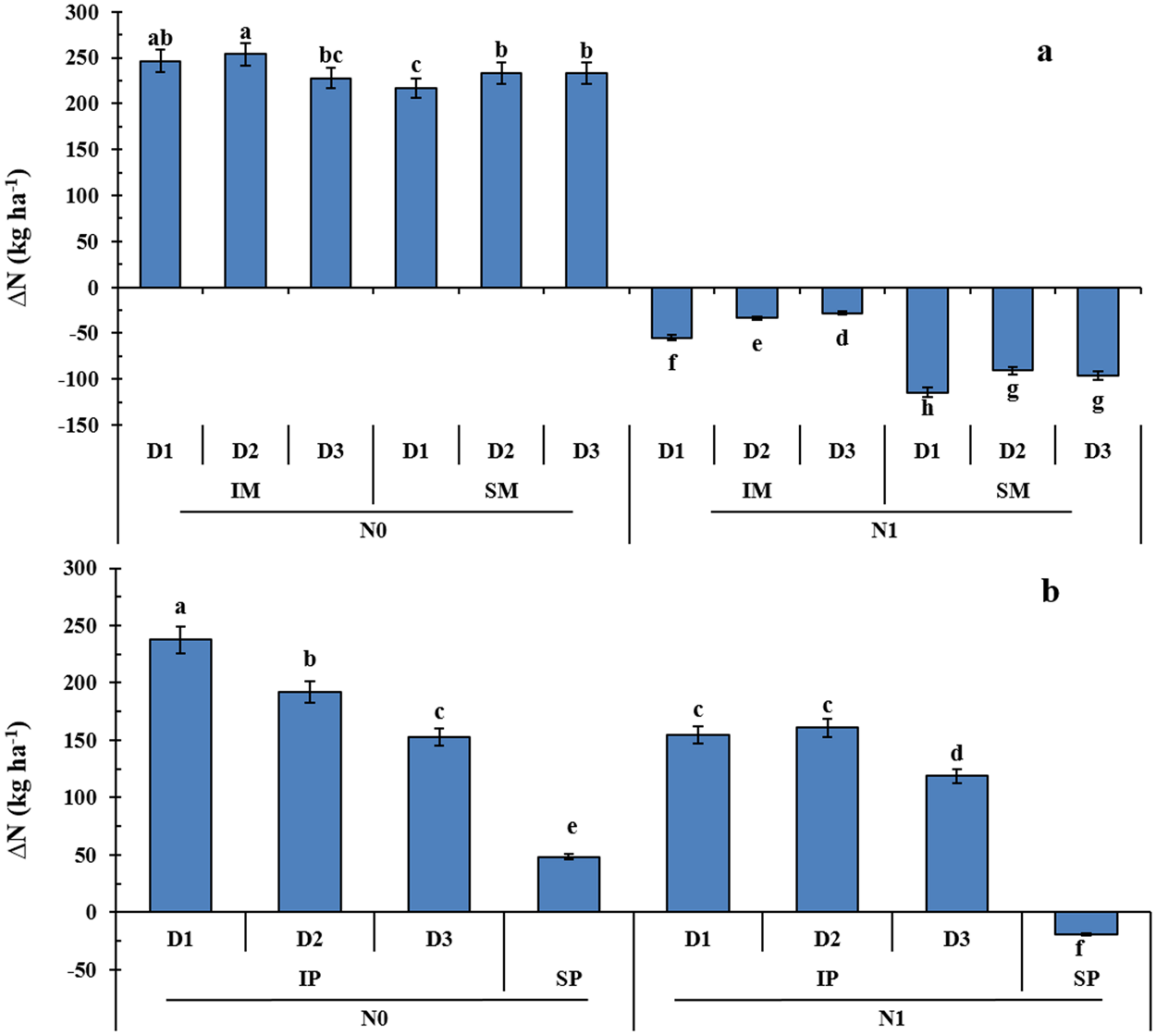
Balance between crop N demand and N supply (∆N) during the growing period of intercropped pea (IP) or sole pea (SP) (**a**) and intercropped maize (IM) or sole maize (SM) (**b**) with two N treatments (N0, 0 kg N ha^−1^; and N1, 225 kg N ha^−1^ for pea and 450 kg N ha^−1^ for maize) and three maize three maize densities (D1, 73,600 plants ha^−1^; D2, 85,900 plants ha^−1^; and D3, 98,200 plants ha^−1^ for sole maize; the relative density of intercropped maize (according to the proportion of occupied area in intercropping) was 45,000, 52,500, and 60,000 plants ha^−1^ for D1, D2, and D3, respectively). Error bars indicate standard errors of the means (*n* = 8 for (**a**), *n* = 12 for (**b**)). Dissimilar letters on bars means that are significantly different according to Fisher’s protected LSD (*P* ≤ 0.05).

Cropping system, N treatment, and maize plant density significantly influenced the balance between crop N demand and N supply (∆N) during the growing season (Fig. 5). The value of ∆N of sole maize with applied N was negative (Fig. 5a). With applied N, intercropped maize exhibited a strongly enhanced N demand compared to that of sole maize. The difference between ∆N of sole maize in D2 and D3 was not significant. However, the ∆N of intercropped maize was increased by 39 and 16% as maize plant density increased from D1 to D2 and from D2 to D3, respectively. The N1 treatment restrained ∆N in pea and maize strips compared to the N0 treatment. With applied N, the average ∆N for intercropped pea decreased by 34% compared to sole pea (Fig. 5b). The ∆N of intercropped pea decreased by 21 and 24% as maize plant density increased from D1 to D2 and from D2 to D3, respectively.

### Grain yield, biomass, and NUE of intercrops

According to the land equivalent ratio (LER) of grain and biomass, intercropping increased total grain yield by 24–30% (Table 3) and biomass of maize and pea by 11–23% compared to sole cropping (Table 4). The LER for grain yield (LER_grain_) of maize and pea intercropped with D3 was 4.8 and 5.2% greater, respectively than that with D1 and D2. Additionally, the LER for biomass (LER_biomass_) of maize and pea intercropped with D3 was 3.9 and 5.3% greater, respectively, than that with D1 and D2.

**Table 3:** Performance of grain yield of intercropped pea and maize relative to corresponding sole crops with two N treatments (N0, 0 kg N ha^−1^; and N1, 225 kg N ha^−1^ for pea and 450 kg N ha^−1^ for maize) and three maize densities (D1, 73,600 plants ha^−1^; D2, 85,900 plants ha^−1^; and D3, 98,200 plants ha^−1^ for sole maize; the relative density of intercropped maize (according to the proportion of occupied area in intercropping) was 45,000, 52,500, and 60,000 plants ha^−1^ for D1, D2, and D3, respectively)^d^.

Nitrogen rates	Maize density	Sole cropping		Intercropping			LER^a^_grain_
		Pea	Maize	Pea	Maize	Total	
		kg ha^−1^					
N0	D1	3275	9459	1827	6511	8338	1.25 ± 0.03^b^
	D2	—	10273	1783	9025	10808	1.26 ± 0.04
	D3	—	11577	1713	9231	10944	1.32 ± 0.03
N1	D1	4679	10499	2188	8086	10274	1.24 ± 0.02
	D2	—	12228	2083	9638	11855	1.24 ± 0.03
	D3	—	12219	1890	10916	12806	1.30 ± 0.02
LSD_(0.05)_		652	989	185	386	862	
		*P* > *F*					
Year (Y)		NS^c^	NS	NS	NS	NS	NS
Nitrogen rate (N)		0.001	0.043	0.021	0.001	0.003	NS
Maize density (D)		—	0.029	0.002	0.041	0.029	0.047
N × D		—	NS	NS	0.038	0.042	NS

^a^LER, land equivalent ratio. ^b^Values following ± are standard errors. ^c^NS, not significant at the 0.05 probability level. ^d^Values are means (*n* = 3).

**Table 4:** Performance of biomass of intercropped pea and maize relative to corresponding sole crops with two N treatments (N0, 0 kg N ha^−1^; and N1, 225 kg N ha^−1^ for pea and 450 kg N ha^−1^ for maize) and three maize densities (D1, 73,600 plants ha^−1^; D2, 85,900 plants ha^−1^; and D3, 98,200 plants ha^−1^ for sole maize; the relative density of intercropped maize (according to the proportion of occupied area in intercropping) was 45,000, 52,500, and 60,000 plants ha^−1^ for D1, D2, and D3, respectively)^d^.

Nitrogen rates	Maize density	Sole cropping		Intercropping			LER^a^_biomass_
		Pea	Maize	Pea	Maize	Total	
		kg ha^−1^					
N0	D1	8798	16970	4130	10854	14984	1.11 ± 0.04^b^
	D2	—	19236	3990	14109	18099	1.13 ± 0.02
	D3	—	20183	3588	15457	19045	1.18 ± 0.02
N1	D1	10069	21061	4860	14707	19568	1.18 ± 0.01
	D2	—	23991	4738	17153	21891	1.19 ± 0.01
	D3	—	23652	4067	19435	23503	1.23 ± 0.02
LSD_(0.05)_		849	1147	376	1370	1056	
		*P* > *F*					
Year (Y)		NS^c^	NS	NS	NS	NS	NS
Nitrogen rate (N)		0.001	0.003	0.001	0.013	0.002	NS
Maize density (D)		—	0.027	0.002	0.014	0.029	0.049
N × D		—	NS	NS	NS	NS	NS

^a^LER, land equivalent ratio. ^b^Values following ± are standard errors. ^c^NS, not significant at the 0.05 probability level. ^d^Values are means (*n* = 3).

Significant grain yield (Table 3) and biomass (Table 4) differences were observed between intercropped and sole maize. The proportion of occupied area in intercropping by maize was 58%. Averaged over years, N treatments, and plant density treatments, maize grain yield and biomass were 80 and 72% of that of sole maize, respectively. Maize grain yield and biomass increased by 14 and 25%, respectively, with N1 compared to N0. Grain yield and biomass of sole maize with D2 were greater than that with D1, but they were not increased as plant density increased from D2 to D3. However, with the N1 treatment, grain yield and biomass of maize increased as plant density increased. Grain yield and biomass of intercropped maize increased by 19 and 17%, respectively, as maize density increased from D1 to D2, and by 13 and 14%, respectively, as the maize density increased from D2 to D3. The proportion of occupied area in intercropping by pea was 42%. Grain yield and biomass of intercropped pea were 49 and 45% of that of sole pea, respectively. Pea grain yield and biomass increased by 22 and 16% with N1 compared to N0. In contrast with maize, grain yield and biomass of pea decreased as maize plant density increased. With the N1 treatment, grain yield of intercropped pea decreased by 5 and 9% as maize density increased from D1to D3 and from D2 to D3, respectively. Similarly, biomass of intercropped pea was reduced by 3 and 14% as maize plant density increased from D1 to D2 and from D2 to D3, respectively. With the N1 treatment, total grain yield and biomass of maize/pea intercropping were greater with increased maize plant density.

**Figure 6:**
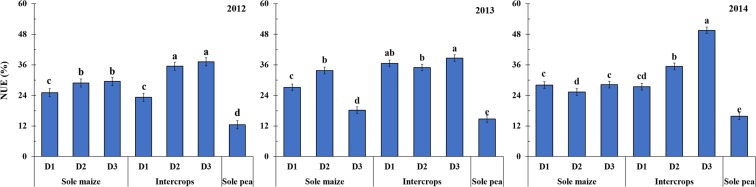
Apparent nitrogen use efficiency (NUE) of intercrops (maize and pea), sole maize, and sole pea from 2012 to 2014 with two N treatments (N0, 0 kg N ha^−1^; and N1, 225 kg N ha^−1^ for pea and 450 kg N ha^−1^ for maize) and three maize three maize densities (D1, 73,600 plants ha^−1^; D2, 85,900 plants ha^−1^; and D3, 98,200 plants ha^−1^ for sole maize; the relative density of intercropped maize (according to the proportion of occupied area in intercropping) was 45,000, 52,500, and 60,000 plants ha^−1^ for D1, D2, and D3, respectively). Error bars indicate standard errors of the means (*n* = 7). Dissimilar letters on bars means that are significantly different according to Fisher’s protected LSD (*P* ≤ 0.05).

Maize/pea intercropping resulted in greater NUE than the corresponding sole cropping systems (Fig. 6). With the D2 treatment, NUE of maize/pea intercrops was 4–40% greater than that of sole maize and 124–184% greater than that of sole pea. With the D3 treatment, NUE of maize/pea intercrops was 26–113% greater than that of sole maize and 162–213% greater than that of sole pea. The NUE of maize/pea intercrops increased as maize plant density increased. The NUE of intercrops increased by 53% in 2012 and 29% in 2014 as maize density increased from D1 to D2, and by 5% in 2012, 11% in 2013, and 40% in 2014 as maize density increased from D2 to D3.

## Discussion

The intensity of crop competition for soil N was significantly affected by soil N supply and plant density^23^. In this study, maize/pea intercropping resulted in interspecific complementary use of soil mineral N by crops. Maize intercropped with pea had greater soil mineral N than sole maize during maize growth after pea harvest. Additionally, less soil mineral N in intercropped pea compared to sole pea during the co-growth period forced pea to rely on N_2_ fixation. In intercropping systems, nutrient uptake of the dominant or early crop is increased during the co-growth stage because of interspecific interaction; however, after the dominant species or early crop is harvested, the subordinate or later crop undergoes a recovery or complementary process so that the final yield is equivalent or greater compared to corresponding solely planted species^24^. Intercropping with increased cereal density increased the competition indices^25^. This might be because cereal species with deep roots have strong N acquisition ability and are able to extend their roots into the neighboring legume strips to acquire more N^26^. In the present study, decreased soil mineral N in both intercropped pea and maize strips with increasing maize density indicates that increasing maize density enhanced the interspecific interaction for improving N uptake by intercrops.

Intercropping alters the N uptake rates of maize and legumes^27^. In the present study, maize/pea intercropping exhibited a greater advantage for total N accumulation in crops compared with the corresponding sole cropping systems. The total NER for maize/pea intercrops ranged from 1.15 to 1.29, except for the treatment combination of N0 and D3. These results indicate that maize and pea had greater N complementation than competition in this intercropping system. Moreover, the variation in partial NER for maize and pea with increasing maize plant density indicates that the competition of maize for N relative to that of pea was promoted by increasing maize density. With increasing maize density, the slight reduction in N accumulation of intercropped pea was compensated by the considerable increase in N accumulation of maize.

The %N_dfa_ of legumes has been shown to increase with greater competition intensity between legume and cereal intercrops, which may be because N concentration in the legume rhizosphere is reduced by the nonlegume^28^. Conversely, in the present study, a significant difference in the %N_dfa_ between intercropped and sole pea was not observed. The %N_dfa_ of pea was significantly decreased with N1 compared to N0, which might be because symbiotic N_2_ fixation is typically inhibited by N fertilization since it increases soil mineral N^29^. Intercropped pea exhibited an increasing trend in %N_dfa_ and a decreasing trend in N_dfa_ with increased maize density. This is because biomass, rather than %N_dfa_, was more critical for the legume to increase N_dfa_^30^. The amount of symbiotically fixed N_2_ by pea was significantly correlated with its biomass.

When N supply (including fertilizer applications and organic matter mineralization) exceeds crop N demand, the excess soil N is susceptible to loss the environment^6^. Improving the balance between crop N demand and supply, a quantitative synchrony, is key to increasing NUE and sustainability in agroecosystems^31,32^. Intercropping with high maize density increased crop N demand, reducing soil mineral N and potential losses of soil N from excessive N fertilization. A high density of cereals enhances the intensity of competition for soil N, which promotes N uptake by crops and reduces mineral N remaining in the soil^33,34^. In the present study, the values of ∆N in pea strips were positive, while those in sole maize with N application were negative. Nitrogen fertilization decreased the ∆N of pea and maize in intercropping or sole cropping systems. In sole maize with N application, N demand (total N accumulation) was substantially lower than N supply (residual soil N available at planting plus N supplied through fertilizer, minus residual soil N at crop harvest). Some of this excess N may be lost due to leaching, denitrification, or ammonia volatilization. When maize or pea were grown without N fertilization, their N demands were largely greater than N supply. Overall, intercropping can significantly improve the balance between crop N demand and N supply compared to sole cropping. Increasing the density of maize is a useful way to improve the balance between N supply and crop demand in maize/pea intercropping with N fertilization.

Intercropping typically increases land productivity and resource use efficiency^35–37^. In the present study, an advantage of intercropping in land productivity and N use according to LER and NER was observed compared to sole cropping. Moreover, the D3 treatment produced greater LER_grain_ and NUE than did D1 and D2. Intercropping reduced the growth of symbiotic legumes via the overshadowing effects of the cereal partner^38^. The plant density in intercropping was a proportion of the density in sole cropping (relative density was 0.58 for maize and 0.42 for pea). Grain yield of intercropped pea was 49% of sole pea, which was greater the proportion of pea occupied area and relative density (42%) in intercropping. This indicates that grain yield of pea was improved by intercropping in pure occupied area compared to sole pea. This may be due to the earlier seedling emergence of pea compared to maize (approximately 15 d). A recent study found that N addition did not increase total grain yield of pea/barley (*Hordeum vulgare* L.) intercropping and decreased the contribution of pea^33^. Other studies have found that grain yield of soybean [*Glycine max* (L.) Merr] in maize/soybean intercropping was increased by 24% with N application compared to that without N application^39^. In the present study, grain yield and biomass of maize and pea were greater with N application compared to without N application. Hybrid characteristics, along with soil and climate conditions, are important factor determining optimal maize plant density^40^. In intercropping, harsh environmental conditions may be ameliorated and the availability of resources may be increased for intercrops^41^. Furthermore, intercropping increases yield of its component crops as it uses soil nutrients more efficiently than sole cropping^42^. Therefore, higher plant density may be adopted with intercropping compared to sole cropping. A previous study on intercropping found that increasing maize plant density reduced legume grain yield and did not increase maize grain yield^43^. In the present study, D3 reduced intercropped pea grain yield compared with D1 and D2, consistent with the trend in biomass, but the increased grain yield of maize with increasing density compensated for the loss of grain yield of intercropped pea. Total grain yield and biomass of maize/pea intercropping were increased as maize plant density increased.

## Conclusions

In conclusion, maize/pea intercropping increases land productivity and NUE than sole cropping. The balance between N supply (with high N fertilizer application) and crop demand was improved with the D3 treatment in maize/pea intercropping. Increasing maize plant density enhanced crop N demand and decreased the soil mineral N at harvest, which reduced potential losses of soil N. Overall, increased maize density is recommended to improve the quantitative synchrony between crop N demand and N supply, thus increasing productivity and NUE of maize/pea intercropping. Further research should be aimed at enhancing the temporal synchrony, as well as quantitative synchrony between N supply and crop demand in order to improve use of N resources and enhance environmental sustainability.

## Materials and Methods

### Experimental site

**Figure 7:**
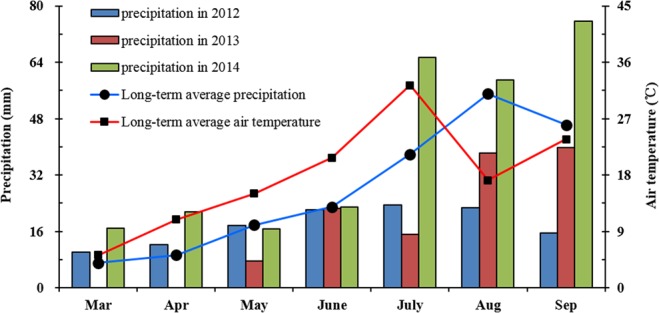
Monthly precipitation during the growing season (1 March to 30 September) in 2012, 2013, and 2014, and long-term average (2005–2014) monthly precipitation and average air temperature at the Oasis Experimental Station located in Wuwei, Gansu province of northwestern China.

A field experiment was conducted in 2012, 2013, and 2014 at the Oasis Agricultural Experiment Station (37°30′N, 103°5′E; 1776 m a.s.l.) of Gansu Agricultural University, located in Wuwei, Gansu Province of northwestern China. The experimental site is located in the temperate arid zone of the Eurasian continent. At this site, the long-term (1950–2014) average annual air temperature is 7.2 °C, with an accumulated air temperature above 10 °C of 2985 °C. Annual precipitation is rarely greater than 155 mm, mainly occurring from June to September, and potential evaporation is 2400 mm. Weather conditions for the three experimental years were near the recent 10-year (2005–2014) average (Fig. 7). The soil at the experimental site is a calcareous Aridisol^44^. The initial main chemical properties of the 0–20 and 20–40 cm soil layers at the experimental site are shown in Table 5. Bulk density samples were collected from three randomly selected locations in each plot using a steel corer (with a volume of 98.12 cm^3^). Soil organic carbon was measured by the Walkley and Black dichromate oxidation method^45^. Total N concentration of soil samples was measured by the dry combustion method using a high-induction furnace C and N analyzer (Elementar vario MACRO cube, Germany). Exchangeable ammonium-nitrogen (NH_4_-N) and extractable nitrate-nitrogen (NO_3_-N) concentrations in the soil were analyzed by a continuous flow analyzer (Autoanalyzer 3, Bran-Luebbe, Germany). Available potassium (K) and phosphorus (P) in the soil were measured according to Zhou *et al*.^46^. The content of soil organic C, total N, mineral N (NH_4_-N + NO_3_-N), available K, and available P was calculated as the product of their concentration and the respective bulk density and thickness for a given soil layer. Soil pH was measured in a 1:2 (m:v) soil solution with 0.01 mol L^−1^ CaCl_2_, using a pH meter (Orion, Thermo Fisher Scientific Inc., Waltham, MA, USA)^47^.

**Table 5:** Bulk density and chemical properties of the 0–20 cm and 20–40 cm soil layers sampled from the experimental field at planting and before fertilizer application in 2012, 2013, and 2014.

Year	Layer (cm)	Bulk density (g cm^−3^)	Organic C	Total N	Mineral N (kg ha^−1^)		Available K	Available P	pH
			(Mg ha^−1^)		NO_3_-N	NH_4_-N	(kg ha^−1^)	(kg ha^−1^)	
2012	0–20	1.48	31	2.4	46	14.5	527	93	7.4
	20–40	1.36	26	2.2	41	13.2	349	77	7.7
2013	0–20	1.43	30	2.6	46	13.6	524	77	7.4
	20–40	1.40	28	2.2	40	12.9	407	60	7.7
2014	0–20	1.41	29	2.2	43	12.7	506	74	7.4
	20–40	1.57	28	2.1	41	12.2	379	67	7.7

### Experimental design

The experimental design was a randomized complete block with three replications. Maize was planted in 2011 prior to this experiment. The treatments were applied to the same plots in each year. Three cropping systems (sole maize, sole pea, and maize/pea intercropping), three maize densities, and two N application rates were investigated. The intercropping was a replacement design. Maize density varied and a constant pea density was used in the intercropping system. The three sole maize densities were 73,600, 85,900, and 98,200 plants ha^−1^ for D1, D2, and D3, respectively. According to the occupied area proportions (occupied area proportions of maize and pea were 58% and 42%, respectively), the relative density of intercropped maize was 58% of sole maize. The densities of intercropped maize were 45,000, 52,500, and 60,000 plants ha^−1^ for D1, D2, and D3, respectively. The density of sole pea was 900,000 plants ha^−1^, and that of intercropped pea was 380,000 plants ha^−1^. Therefore, maize in intercropping and sole cropping had equal densities of pure occupied area. The two N treatments were N0 (0 kg N ha^−1^ for maize and pea) and N1 (450 kg N ha^−1^ for maize and 225 kg N ha^−1^ for pea). For the N1 treatment, 225 kg N ha^−1^ as ammonium nitrate was broadcast and incorporated into the top 20 cm of the soil as a base fertilizer prior to the planting maize and pea. Then, 112.5 kg N ha^−1^ as urea was top-dressed at both the 6- and 12-leaf stages of maize phenological development^48^.

**Figure 8:**
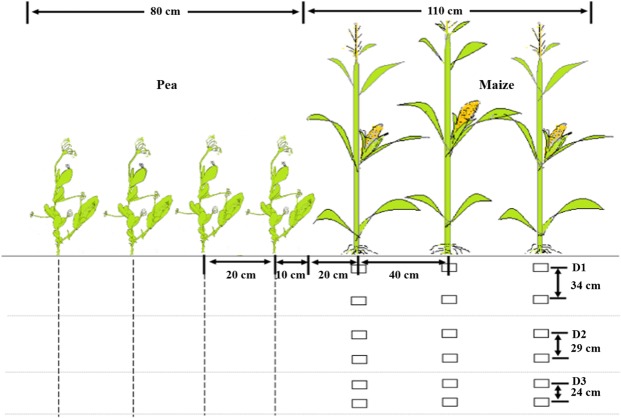
Strip structure of maize/pea intercropping and field locations where soil samples were collected annually. Each maize strip (110 cm wide) consisted of three rows of maize spaced 40 cm apart, and each pea strip (80 cm wide) consisted of four rows of pea spaced 20 cm apart. Sole maize treatments included three plant densities controlled by interplant distance: D1 at 73,600 plants ha^−1^, D2 at 85,900 plants ha^−1^, and D3 at 98,200 plants ha^−1^; the relative density of intercropped maize (according to the proportion of occupied area in intercropping) was 45,000, 52,500, and 60,000 plants ha^−1^ for D1, D2, and D3, respectively.

In the intercropping treatment, one strip (1.9 m wide) included three maize rows and four pea rows, and one plot contained three strips (Fig. 8). The inter-row spacing was 40 cm for maize and 20 cm for pea, and the distance between the maize row and the nearest pea row was 0.30 m in the intercropping treatment. All plots were 8 m × 5.7 m, with a 0.5 m wide ridge surrounding each plot. The dates of planting were 1 Apr. 2012, 2 Apr. 2013, and 1 Apr. 2014 for pea (cultivar Long-wan no.1) and 21 Apr. 2012, 22 Apr. 2013, and 20 Apr. 2014 for maize (cultivar Xian-yu 335). All plots received 150 kg P_2_O_5_ ha^−1^ prior to planting at the time of the first N application, using calcium superphosphate (0–16–0 of N-P_2_O_5_-K_2_O). Irrigation was applied to all plots during the growing season to prevent water stress (Table 6). Irrigation was applied using pipes (13 cm diameter), and a water meter installed at the discharging end of the pipe was used to determine the amount of water applied.

**Table 6:** Irrigation schedule and amounts of water applied to the cropping system treatments in this experiment in each of the three years.

Treatment	Maize 2-leaf stage	Maize 6-leaf stage	Maize 12-leaf stage	Maize silking stage	Maize Kernel milk stage	Irrigation total
	mm					
Sole pea	90	120	—	—	—	210
Sole maize	90	120	120	120	90	540
Intercropping	90	120	120	120	90	540

The irrigation schedule is based on maize phenological development^48^.

### Sample collection and analysis

Soil samples were collected from the 0–20, 20–40, 40–60, 60–90, and 90–120 cm soil layers before planting and at harvest of pea or maize using a soil drill made of an iron tube (150 cm length × 6.5 cm internal diameter × 7.0 cm external diameter). During the growing season, soil samples were collected every 15 d before harvest of pea and every 20 d from pea harvest to maize harvest; samples were divided into 0–20 and 20–40 cm depth increments. Four soil cores were collected from the intercropping system (Fig. 2) and mixed thoroughly to obtain a composite sample. Two cores were obtained from pea rows and two cores were obtained from maize rows. For the sole crop treatments, two soil cores were collected from each plot and mixed thoroughly. Soil cores were passed through a 2 mm sieve and then extracted with 100 mL of 0.01 mol L^−1^ CaCl_2_ to determine soil mineral N (NH_4_-N and NO_3_-N) concentration using a continuous flow analyzer (Autoanalyzer 3, Bran-Luebbe, Germany).

Ten maize plants and four adjacent rows of pea, with row lengths of 50 cm, were sampled from each plot. Plant samples were collected every 15 d before pea harvest and every 20 d from pea harvest to maize harvest. Maize and pea biomass were measured by oven drying samples at 80 °C to a constant mass and weighing. Maize and pea plant samples were milled after oven drying for analysis of N and δ^15^N. Nitrogen concentration in plant biomass was determined by a high-induction furnace C and N analyzer (Elementar vario MACRO cube, Germany) after oven drying and grinding to 1 mm. Mass spectrometry (DELTAplus XP, Thermo Finnigan, Bremen, Germany) was used to determine δ^15^N. At crop physiological maturity, plots were harvested by hand and the harvested grain was air-dried, cleaned, and weighed to determine grain yield.

### Calculations

During the growing season, mineral N (NO_3_-N and NH_4_-N) in the 0–20 cm and 20–40 cm soil layers was calculated as the product of bulk density and concentration of soil NO_3_^–^-N or NH_4_^+^-N. At planting and harvest of pea and maize, mineral N in the soil profile (0–120 cm) was also calculated as:

1 $${\rm{Y}}={{\rm{\Sigma }}{\rm{T}}}_{{\rm{i}}}{{\rm{BD}}}_{{\rm{i}}}{[{{\rm{NO}}}_{3}^{-}]}_{{\rm{i}}}+{{\rm{\Sigma }}{\rm{T}}}_{{\rm{i}}}{{\rm{BD}}}_{{\rm{i}}}{[{{\rm{NH}}}_{4}^{+}]}_{{\rm{i}}}$$

where T_i_, BDi, [NO_3_^−^]_i_, and [NH_4_^+^]_i_ are the thickness, bulk density, and concentration of soil NO_3_^−^ and NH_4_^+^ in a given soil layer, respectively^49^.

The percentage of total aboveground N accumulation for pea, derived from N_2_-fixation (%N_dfa_), was calculated using the ^15^N content of pea (δ^15^N_pea_) and maize (δ^15^N_maize_):

2 $$ \% {{\rm{N}}}_{{\rm{dfa}}}=100\times \frac{{{\rm{\delta }}}^{{\rm{15}}}{{\rm{N}}}_{{\rm{maize}}}-{{\rm{\delta }}}^{{\rm{15}}}{{\rm{N}}}_{{\rm{pea}}}}{{{\rm{\delta }}}^{{\rm{15}}}{{\rm{N}}}_{{\rm{maize}}}-{\rm{B}}}$$

where δ^15^N_pea_ and δ^15^N_maize_ represent δ^15^N (‰) values of pea and maize growing in the same field. The δ^15^N (‰) values were calculated based on the ^15^N natural abundance method^50^. The B value for pea (−1.05) was derived from δ^15^N natural abundance analysis of pea grown with N-free sand^49^.

Crop N derived from the atmosphere (N_dfa_) was calculated based on crop biomass yield and N concentration (%N):

3 $${{\rm{N}}}_{{\rm{dfa}}}={\rm{Biomass}}\times  \% {\rm{N}}\times (\frac{ \% \mathrm{Ndfa}}{{\rm{100}}})$$

Nitrogen accumulation by crops was calculated as the product of biomass and N content.

The ∆N was used to determine the balance between crop N demand and N supply during the growing season:

4 $${\rm{\Delta }}N={{\rm{N}}}_{{\rm{accumulation}}}\mbox{--}{({\rm{N}}}_{{\rm{planting}}}+{{\rm{N}}}_{{\rm{fertilizer}}}\mbox{--}{{\rm{N}}}_{{\rm{harvest}}})$$

where ∆N is the balance between crop N demand and N supply during the growing season, N_accumulation_ is the total aboveground N accumulation in plants, N_planting_ and N_harvest_ are soil N measured at planting and harvest, respectively, and N_fertilizer_ is N derived from fertilizer applied during the growing season. A ∆N of zero indicates that N demand of the cropping system (N accumulation in crops) was equal to N supply (residual soil N at planting plus fertilizer N, minus residual soil N at crop harvest). A positive ∆N indicates that N demand of the cropping system exceeded the N supply and that some N may be from soil N mineralization or N_2_ fixation. A negative ∆N indicates that N demand of the cropping system was less than N supply and that excess N may be susceptible to loss through leaching, denitrification, or ammonia volatilization.

The total land equivalent ratio (LER) was used to evaluate the relative advantage of intercropping compared to sole cropping^51^. This index expresses the land area required under sole cropping to produce the yields obtained in intercropping. The N equivalent ratio (NER), LER for grain yield (LER_grain_), and biomass (LER_biomass_) for intercropping of pea and maize were calculated according to Rao and Willey^51^. Total LER is the sum of partial NER and LER values for pea and maize: NER_pea_ or LER_pea_ = (Y_IP_ × A_P_)/Y_SP_, NER_maize_ or LER_maize_ = (Y_IM_ × A_M_)/Y_SM_, total NER or LER = LER_pea_ + LER_maize_, where Y_IP_ and Y_SP_ are N accumulation, grain yield, or biomass of intercropped pea and sole pea, respectively, Y_IM_ and Y_SM_ are N accumulation, grain yield, or biomass of intercropped maize and sole maize, respectively, and A_P_ and A_M_ are the ratios of area occupied by pea and maize in the intercropping system relative to that of the corresponding sole cropping systems, respectively.

Apparent N use efficiency (NUE) from treatments with N applied was calculated according to Dalal *et al*.^52^:

5 $${\rm{NUE}}( \% )=100\times \frac{{\rm{N}}\,{\rm{uptake}}\,{\rm{from}}\,{\rm{N}}\,{\rm{treatment}}\,-\,{\rm{N}}\,{\rm{uptake}}\,{\rm{from}}\,{\rm{control}}}{{\rm{N}}\,{\rm{applied}}}$$

### Statistical analysis

Statistical analysis was conducted at *P* ≤ 0.05 using the SPSS software package (version 19.0, SPSS, Chicago, USA). The year × treatment interaction and the main effects of year and treatment were assessed using ANOVA. When a significant year × treatment interaction was not observed, the three years of data were pooled for mean comparisons. Means were compared using Fisher’s protected LSD test at *P* ≤ 0.05.
